# Balloon dilation and rapid maxillary expansion: a novel combination treatment for congenital nasal pyriform aperture stenosis in an infant

**DOI:** 10.1186/s13052-021-01124-2

**Published:** 2021-09-16

**Authors:** Emanuela Sitzia, Sara Santarsiero, Filippo Maria Tucci, Giovanni De Vincentiis, Angela Galeotti, Paola Festa

**Affiliations:** 1 Unit of Otolaryngology, Bambino Gesù Children’s Hospital, 00165 Rome, Italy; 2 Unit of Dentistry, Bambino Gesù Children’s Hospital, 00165 Rome, Italy

**Keywords:** Pyriform aperture stenosis, CNPAS, PAS, Balloon dilation, Palatal expander, Nasal endoscopy, Infant nasal obstruction, Congenital nasal stenosis, Craniofacial anomalies, Obstructive sleep apnoea syndrome

## Abstract

**Background:**

Congenital nasal pyriform aperture stenosis (CNPAS) is a rare condition that may occur alone or as part of a multi-formative syndrome. Management remains difficult. There is no specific treatment protocol. Traditional surgery would be anachronistic; a non-invasive or minimally invasive therapeutic option is required. However, the rarity of the disease and the infantile context render randomised clinical trials difficult.

**Case presentation:**

We present the case of a one-month-old Caucasian boy with CNPAS. He presented to the Emergency Department of the Bambino Gesù Pediatric Hospital with nasal obstruction, noisy breathing, feeding difficulties, and suspected sleep apnoea. During hospitalisation, he underwent overnight pulse oximetry, airway endoscopy, and maxillofacial computed tomography (CT); the final diagnosis was CNPAS with moderate obstructive sleep apnoea syndrome. We successfully treated the patient using an innovative strategy that involved collaboration between ear-nose-and-throat surgeons and orthodontists.

**Conclusions:**

A combination of minimally invasive balloon surgery and placement of a palatal device may successfully treat CNPAS; it may also treat other types of nasal bone stenosis. Future studies may allow the development of practice consensus treatment strategies.

## Background

Nasal obstruction is a frequent cause of infant morbidity. This is most commonly attributable to mucosal oedema, but other less common pathologies must be considered (e.g., congenital nasal masses and congenital bony stenosis). Depending on the location of the constriction, congenital bony stenosis is divided into anterior (congenital nasal pyriform aperture stenosis, CNPAS), middle (midnasal stenosis), and posterior stenosis (choanal atresia) [1]. First described by radiologists in 1988 and one year later by ear-nose-and-throat specialists [2, 3], CNPAS is a rare condition characterised by narrowing of the nasal cavity at the level of the pyriform aperture, attributable to medial positioning or overgrowth of the maxillary process. CNPAS is frequently associated with other craniofacial or neurological anomalies; the incidence of isolated CNPAS remains unclear [4–7]. The principal pathogenetic theory presumes that CNPAS is an abnormal overgrowth of the nasal maxillary process associated with lateral palatal shelf overlapping during secondary palate fusion in the fourth month of gestation [8]. As in other congenital nasal stenoses, CNPAS morbidity may range from severe respiratory distress requiring immediate oral or orotracheal respiratory assistance to a mild condition that may compromise feeding and thus impair growth. Regardless of severity, early diagnosis is advisable because neonates are obligate nasal breathers and may not tolerate long-term nasal obstruction. Severe nasal obstruction is usually identified at birth by an inability to pass a nasal tube. However, the condition sometimes remains undiagnosed until the onset of symptoms such as noisy breathing, tachypnoea, cyanosis, desaturations, aspiration, difficult feeding, and reduced growth. Acute respiratory distress and cyanosis are typically relieved by crying and tend to return on rest (“paradoxical cyanosis”). The basic physical examination assesses noisy nasal breathing, any reduction in nasal airflow, and any signs of chronic respiratory fatigue. If a congenital nasal obstruction is suspected, nasal endoscopy should be performed to orient the clinician. In patients with CNPAS, passage of a 2.2-mm-diameter flexible laryngoscope is usually halted at the entrances of both nasal cavities. A CNPAS diagnosis must be confirmed via maxillofacial computed tomography (CT) (axial and coronal sections). The width of the piriform aperture normally ranges from 8.8 to 17.2 mm; a measurement < 11 mm at the axial level of the inferior meatus in a term neonate is considered diagnostic of CNPAS by most clinicians [9]. CT usually reveals a bony crest along the median axis of the hard palate (i.e., a median palatal ridge) or a triangular palate. Often, CNPAS co-exists with congenital midnasal stenosis (another rare condition) attributable to unequal growth of the lateral nasal wall or excessive folding of the nasal septum. Other possible CNPAS-associated features evident on CT include abnormal dentition; a central mega-incisor is present in 50% of cases (solitary median maxillary central incisor syndrome). CNPAS combined with solitary median maxillary central incisor syndrome may be part of the holoprosencephaly spectrum. Hypopituitarism may also be apparent. Brain magnetic resonance imaging and genetic counselling are recommended if any neurological or syndromic abnormality is evident [10–12].

Several CNPAS treatments have been proposed based on the severity of nasal respiratory impairment. Mild conditions require medical treatment followed by watchful waiting. The first-level strategies in various studies [13, 14] have included nasal decongestants or corticosteroids, saline irrigation, anti-reflux agents, oxymetazoline hydrochloride, humidifiers, mouth-breathing devices (e.g., McGovern nipple), and non-invasive positive pressure ventilation. If CNPAS is severe, or if no improvement is observed, the current recommendation is surgical correction. The traditional technique features osteotomy of the nasal lateral wall through a sublabial incision drilled with the aid of a loupe, followed by nasal stent placement for 5–28 days [15]. Some clinicians have combined osteotomy with inferior turbinate reduction or resection, without the placement of nasal stents [16]. Although generally effective, this technique may be associated with non-trivial intra- and postoperative complications [17]. Therefore, most clinicians have shifted to less invasive approaches. Examples include placement of a Hegar cervical dilator or airway balloon with or without subsequent nasal stenting [18, 19]. Rapid maxillary expansion (RME) and surgically assisted rapid palatal advancement have been used to enlarge the nasal space via palatal distraction in infants with CNPAS or midnasal stenosis [20–22]. Here, we present the case of an infant with CNPAS who was treated via combined balloon dilation and placement of an innovative (removable) oral device facilitating rapid palatal expansion; we term this the Neonatal Palatal Expander Plate (NPEP).

## Case presentation

A 1-month-old Caucasian boy was referred to the Emergency Care Unit of Bambino Gesù Pediatric Hospital (Palidoro, Rome, Italy) with noisy nasal breathing, nasal obstruction, and suspected respiratory distress. He had been delivered via caesarean section at 38 weeks of gestation, following a normal pregnancy. His birth weight was 3028 g and his APGAR score was 6. He exhibited respiratory distress and mild hypotonus. Positive-end expiratory pressure was initially applied, with a change to continuous positive airway pressure for 20 h, and finally a change to high flow oxygen for 3 days. The neonatologists did not detect any particular problem and the infant was discharged in apparently good condition. Over the following days, the parents noticed that he experienced increased difficulty in nasal breathing during breast-feeding. Despite this, his weight gain rate was within normal limits, but he was very irritable. Awakenings with crying were very frequent. Cyanosis and apnoea were not evident, but his nose appeared to be chronically congested despite daily irrigation and aspiration. The parents did not report any family history of congenital malformations.

**Fig. 1:**
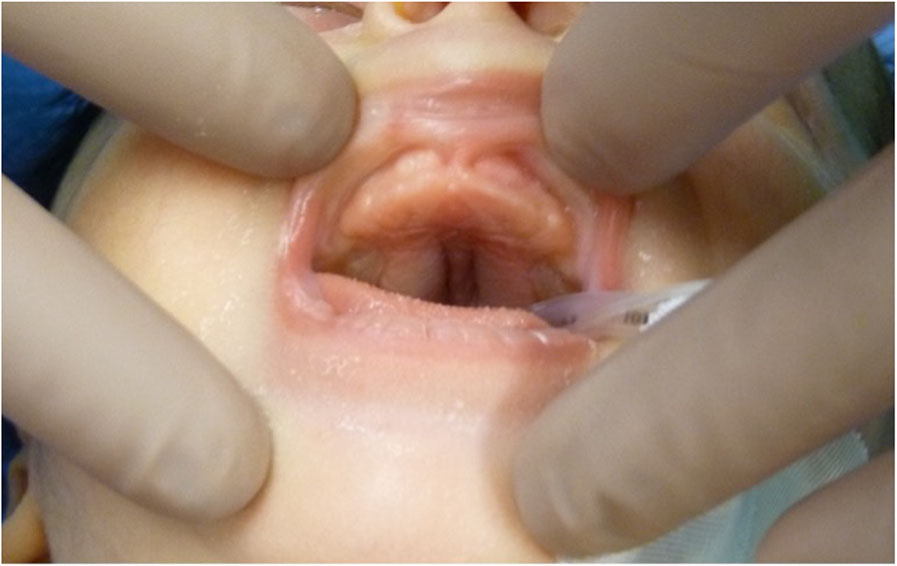
Clinical evaluation before treatment revealed an ogival palate with a noticeable depression in the median palatine raphe

At our first evaluation, the infant’s appearance was rosy and his oxygen saturation was 97% in ambient air. His crying was normal; he exhibited no sign of chronic respiratory distress. Probing of the nasal cavities revealed clinically significant passage impediment, particularly in the right nares. The palate was ogival in nature, with a noticeable depression in the region of the median palatine raphe (Fig. 1). Nasal endoscopy using a 2.2-mm-diameter paediatric flexible endoscope was attempted with the patient awake, but the device could not be advanced.

**Fig. 2:**
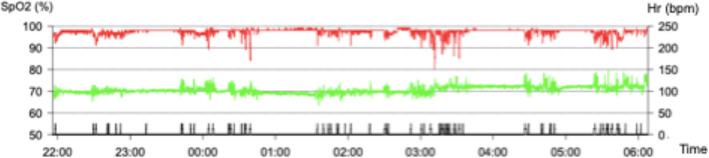
Overnight pulse oximetry performed before treatment showed clusters of desaturation and concomitant increases in heart rate. Moderate obstructive sleep apnoea was evident

**Fig. 3:**
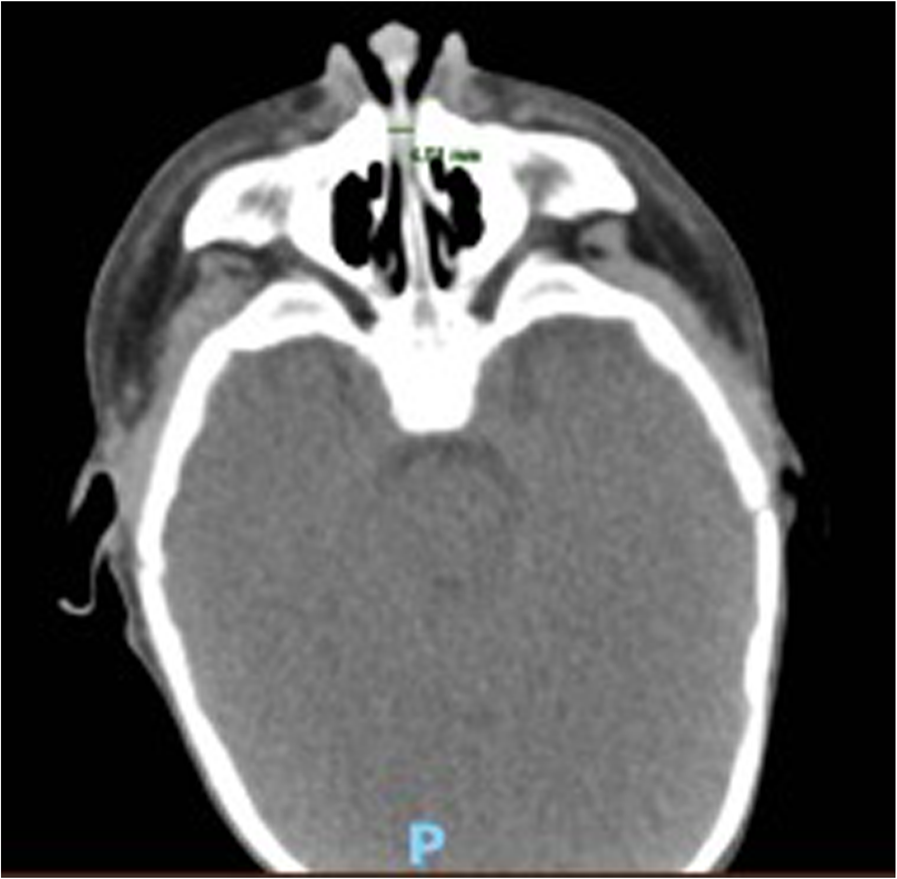
Preoperative head CT revealed pyriform aperture stenosis. The measurement was taken at the axial level of the inferior meatus, as recommended by radiological guidelines

**Fig. 4:**
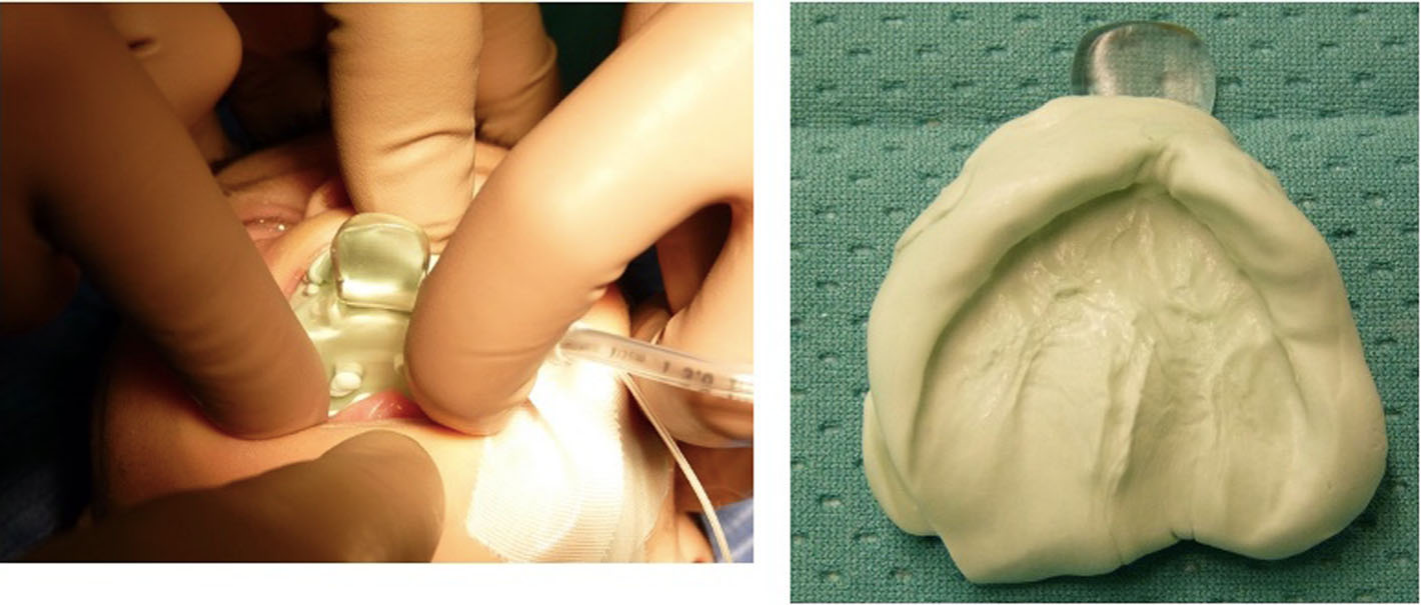
Left (**A**): Impression of the upper jaw obtained during operation. Right (**B**): Impression after addition of silicon

The infant was hospitalised for diagnostic investigations. During his first night, he underwent pulse oximetry to assess the severity of respiratory distress during sleep. His average and minimum oxygen saturation levels were 97.5 and 80%, respectively; his oxygen desaturation index was 8.4. These parameters indicated moderate obstructive sleep apnoea syndrome (Fig. 2). Head CT (Fig. 3) revealed bilateral stenosis of the pyriform aperture, with a transverse diameter of 4.01 mm. The choanal diameter was within the normal range. Thus, we planned complete airway endoscopy with the patient under general anaesthesia, simultaneous balloon dilation of the pyriform stenosis, and collection of an upper jaw impression by a paediatric dentist (Fig. 4A, B).

**Fig. 5:**
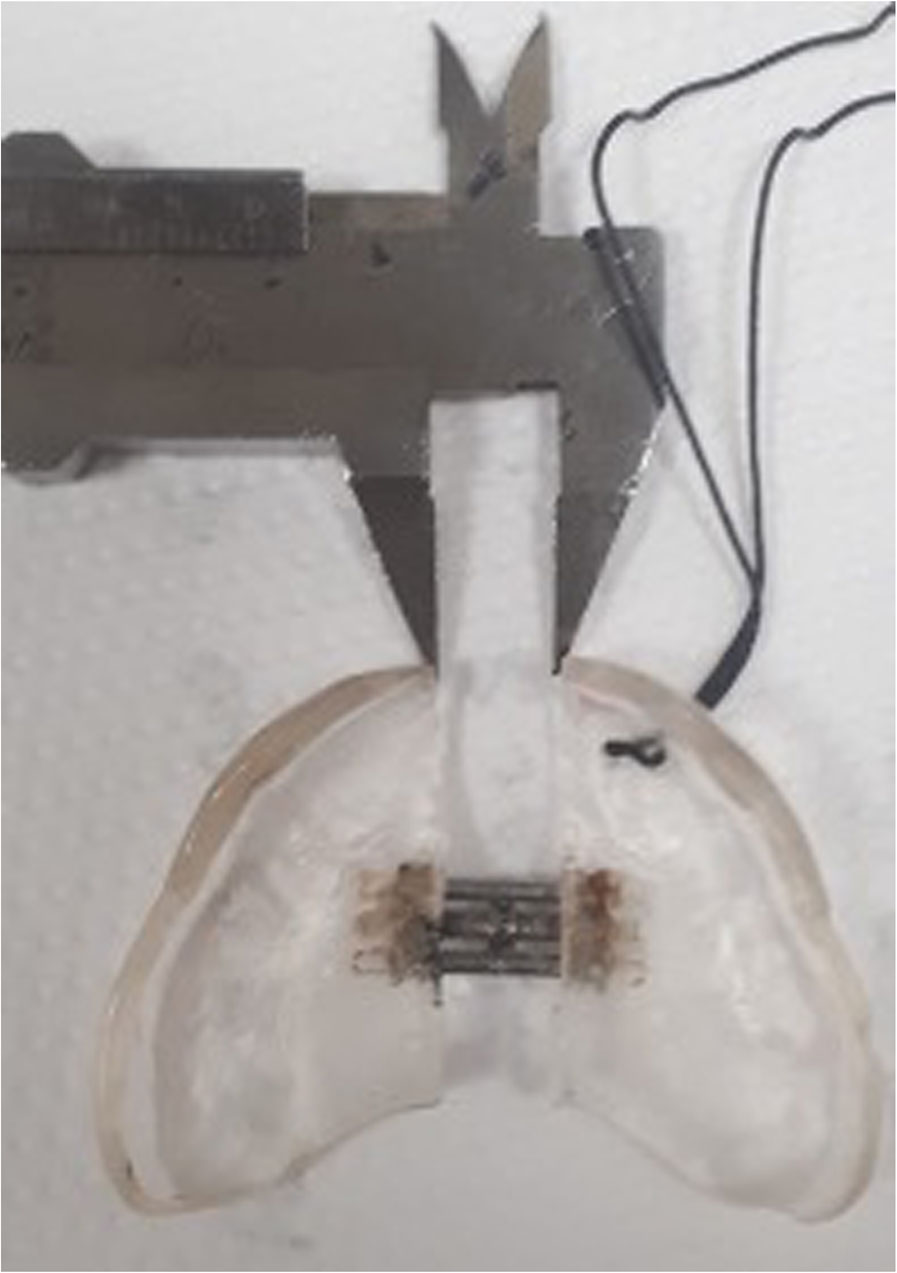
The Neonatal Palatal Expander Plate (NPEP) made of transparent acrylic resin with a 12-mm-long screw (median palatal suture). The total screw expansion was 11.5 mm

Endoscopy confirmed radiological stenosis of the anterior and middle thirds of the nasal cavities; choanal patency; and a normal larynx, trachea, and bronchi. Nasal dilation commenced with the introduction of a 2-mm-diameter Hegar cervical dilator, followed by 3- and 4-mm dilators and an 8-mm airway balloon. The nasal respiratory space increased without any complication. The infant’s parents were instructed to perform daily nasal irrigation with saline. A steroid-containing nasal spray was administered once daily for 1 month to reduce nasal inflammation and discourage restenosis. A removable NPEP was constructed by a dental technician using the upper arch cast. A flanged mucous anchor plate of transparent acrylic resin was extended toward the oral vestibule. A 12-mm-long screw was placed in the centre (median palatal suture). To avoid any risk of suffocation, a safety wire (surgical silk suture thread without a needle, approximately 7 cm in length) was inserted through two holes in the canine region. The device was surgically placed and the parents were instructed to insert it for nearly 14 h per day; they were instructed to turn the screw twice daily for 20 days, then once daily for 12 days. The total screw expansion was 11.5 mm (Fig. 5).

Overnight pulse oximetry and nasal endoscopy were repeated 1 and 2 months later. No clusters of desaturation were evident. The first endoscopy was performed with the infant under general anaesthesia. Patency of the piriform aperture was optimal in the right nasal cavity; in the left nasal cavity, a small non-obstructive bone spur was apparent on the lateral wall. We thus considered it appropriate to perform a second balloon dilation only in the left nasal cavity. The 2-month endoscopic check-up was performed with the infant awake in the outpatient clinic; the findings confirmed complete restoration of patency in both nasal cavities. The parents were instructed to continue to apply the palatal expander for 12 h daily for approximately 30 days. The 6-month evaluation revealed a stable clinical picture. The patient is scheduled for long-term 6-month follow-ups to ensure that craniofacial growth remains harmonious.

## Discussion and conclusions

Treatment of CNPAS and midnasal stenosis should reflect clinical severity in terms of respiratory distress and an inability to feed. If the condition is mild, medical therapy may support the patient until somatic growth triggers spontaneous recovery. In patients who are unresponsive, who worsen, or who exhibit moderate-to-severe disease, anatomical correction is required. The traditional treatment features bony drilling (through a sublabial incision) followed by nasal stenting. This approach is currently considered to be the most effective; it has been successfully used to treat several severe case series [23]. Despite the good results, the risks include injury to the nasal mucosa, septum, nasal ala, nasolacrimal duct, and tooth buds; anomalies may occur in facial mass development and dentition [17, 19]. Septal ulceration occurred in 24% of cases [19]. Furthermore, age-related surgical risks should be considered, especially when CNPAS is associated with other craniofacial anomalies. Efforts have been made to develop less invasive, rapid, and effective long-term therapeutic strategies, as well as to reduce morbidity. Novel approaches use dilation techniques that do not require mucosal incision or bone drilling.

A Hegar cervical dilator and an airway balloon immediately open the bony stenosis; infant maxillofacial bone and cartilage is plastic. The rigid dilator allows application of high initial traction, but may be traumatic if a limit is exceeded. Therefore, dilation was completed using a balloon. Gungor et al. [18] successfully treated a 2-week-old neonate through balloon dilation of the pyriform aperture, followed by nasal stenting for 12 weeks and topical steroids for 2 months. Because stenting may traumatise the nasal mucosa and thus be poorly tolerated in infants, some clinicians [19] have performed dilation without nasal stenting, although they reported subsequent restenosis. Nasal stenting usefully stabilises dilation but is difficult to manage in infants; it is associated with risks of mucosal damage, nasal scarring, and epistaxis. We sought an alternative to stenting through collaboration with orthodontists. In recent decades, management of childhood nasal obstruction and obstructive sleep apnoea syndrome has benefitted from collaborations between dentists and ear-nose-and-throat specialists. RME is routinely used by orthodontists to increase the transverse palatal diameter, facilitating nasal breathing by widening the nasal base [20]. Collares et al. [21] treated an infant with CNPAS by placing a fixed RME device while the patient was under general anaesthesia. Palatal expansion using oral devices has effectively treated congenital nasal stenosis [20, 21] but not severe obstruction. Furthermore, although maxillary remodelling is quicker in infants than in adults, an infant with CNPAS may require immediate restoration of nasal patency; any delay may be associated with morbidity or moderate-to-severe obstruction. The risk of increasing respiratory distress limits the use of RME alone. Surgically assisted rapid palatal advancement (SARPE) achieves palatal distraction through surgical osteotomies and placement of an oral device in patients with crowded maxillary dentition or maxillary hypoplasia. A recent successful application in a case of midnasal stenosis was described by Graham et al. [22], but the authors expressed concern regarding possible long-term risks [22] such as asymmetric expansion, gingival recession, periodontal bone defects with loss of the central incisors, and osteotomy site infections [24].

In our experience, ideal CNPAS (and midnasal stenosis) treatment would combine a mini-invasive approach (ensuring immediate nasal patency through balloon dilation) with slower maxillary expansion to achieve long-term stability. A mobile (not fixed) device can be removed without sedation or general anaesthesia. However, a screw in the maxilla is invasive and not risk-free. A safety cord was placed to allow a parent to perform rapid plate removal if required.

In conclusion, a combination of minimally invasive techniques and collaboration between professionals resulted in therapeutic success. We presume that a similar approach may be useful when encountering other nasal stenoses (e.g., midnasal stenosis). Further studies are needed.

## Data Availability

All data generated or analysed during this study are included in this publication. We cite any publicly available data on which the conclusions rely, including persistent identifiers.

## Abbreviations

CNPAS: congenital nasal pyriform aperture stenosis
RME: rapid maxillary expansion
NPEP: Neonatal Palatal Expander Plate
